# Letter Regarding: Long-term Outcomes of Microfracture for Treatment of Osteochondral Lesions of the Talus

**DOI:** 10.1177/10711007211058693

**Published:** 2022-01-13

**Authors:** Carlijn S. ter Laak Bolk, Jari Dahmen, Gino M.M.J. Kerkhoffs

Dear Editor,

We thank the team of Dr Corr^1^ for performing an interesting study titled “Long-term Outcomes of Microfracture for Treatment of Osteochondral Lesions of the Talus.” Although we read the work with pleasure, some thought-provoking points were raised which we wish to respectfully discuss.

Firstly, 55% of the patients were available for follow-up. Obviously, this introduces risk of bias affecting outcomes and conclusions based on the performed analyses. Despite comparing the included cohort to the lost-to-follow-up cohort, important demographic, prognostic, and outcome factors are lacking in this analysis (eg, lesion size and morphology). Excluding patients with larger lesion sizes or prognostically inferior lesion morphologies can skew the results favorably.

Furthermore, the study’s purpose was to determine whether orthobiologic treatment is a necessary addition to BMS for long-term clinical outcomes. It was not mentioned whether orthobiologics contributed to improving long-term outcomes, thereby not completing the study’s purpose. We wonder whether orthobiologics were used in the current population and whether a subanalysis could have been performed. Moreover, the study did not refer to relevant publications researching long-term clinical outcomes after BMS. Discussing European publications would have contributed to the quality of the discussion as recent publications assessed that the long-term outcomes are less favorable than the authors presented.^2,4,5^ Comparing the results in a more comprehensive manner can yield insights why such promising results were found in a treatment that we think may not do that well at the long term.

Finally, the Foot and Ankle Ability Measure (FAAM) and visual analog scale (VAS) for pain were used. The validity and reliability of the FAAM can be questioned as this score has not (yet) been specifically validated to assess outcomes of OLT treatment. Besides, it is unclear in which setting (rest, standing, during activities) the patients’ pain was assessed, making the interpretation of the VAS scores impossible. This distinction is vital for OLT patients as they will have higher pain scores during weightbearing. Moreover, higher pain scores during weightbearing can imply cartilage or subchondral bone degeneration following microfracture, negatively affecting outcomes in the long term.^3^

Concluding from the above, we can state that the study’s results should be received and interpreted with a number of reservations as the results appear more promising compared to the current literature on the long-term efficacy of BMS. Subsequently, it is yet to be determined whether orthobiologics have a role in improving long-term outcomes.

## Supplemental Material

sj-pdf-1-fai-10.1177_10711007211058693 – Supplemental material for Letter Regarding: Long-term Outcomes of Microfracture for Treatment of Osteochondral Lesions of the Talus Click here for additional data file. Supplemental material, sj-pdf-1-fai-10.1177_10711007211058693 for Letter Regarding: Long-term Outcomes of Microfracture for Treatment of Osteochondral Lesions of the Talus by Carlijn S. ter Laak Bolk, Jari Dahmen and Gino M.M.J. Kerkhoffs in Foot & Ankle International

Carlijn S. ter Laak Bolk, BSc *Department of Orthopedic Surgery, Amsterdam Movement* *Sciences, Amsterdam UMC*, *Location AMC, University of Amsterdam*, *Amsterdam, the Netherlands* *Academic Center for Evidence based Sports medicine (ACES)*, *Amsterdam, the Netherlands* *Amsterdam Collaboration for Health and Safety in Sports (ACHSS)*, *International Olympic Committee (IOC) Research Center, Amsterdam UMC* *Amsterdam, the Netherlands*
Jari Dahmen, MD *Department of Orthopedic Surgery, Amsterdam Movement Sciences, Amsterdam UMC*, *Location AMC, University of Amsterdam*, *Amsterdam, the Netherlands*
*Academic Center for Evidence based Sports medicine (ACES)*, *Amsterdam, the Netherlands*
*Amsterdam Collaboration for Health and Safety in Sports (ACHSS)*, *International Olympic Committee (IOC) Research Center*, *Amsterdam UMC* *Amsterdam, the Netherlands*
Gino M.M.J. Kerkhoffs, MD, PhD *Department of Orthopedic Surgery, Amsterdam Movement Sciences, Amsterdam UMC*, *Location AMC, University of Amsterdam*, *Amsterdam, the Netherlands*
*Academic Center for Evidence based Sports medicine (ACES)*, *Amsterdam, the Netherlands*
*Amsterdam Collaboration for Health and Safety in Sports (ACHSS)*, *International Olympic Committee (IOC) Research Center, Amsterdam UMC*
*Amsterdam, the Netherlands*
*Email: g.m.kerkhoffs@amsterdamumc.nl*

